# Cisplatin-induced genetic alterations in *KEAP1* promote therapeutic resistance in head and neck squamous cell carcinoma

**DOI:** 10.1016/j.redox.2025.103819

**Published:** 2025-08-11

**Authors:** Yuki Nakayama, Keiko Taguchi, Shun Wakamori, Akira Uruno, Akihito Otsuki, Akira Ohkoshi, Hidekazu Shirota, Tomoyuki Iwasaki, Yukio Katori, Masayuki Yamamoto

**Affiliations:** a Department of Biochemistry and Molecular Biology, Tohoku Medical Megabank Organization, Tohoku University, Sendai, Japan; b Department of Otorhinolaryngology-Head and Neck Surgery, Tohoku University Graduate School of Medicine, Sendai, Japan; c Laboratory of Food Chemistry, Department of Applied Biological Chemistry, Graduate School of Agricultural and Life Sciences, The University of Tokyo, Tokyo, Japan; d Department of Medical Oncology, Tohoku University Graduate School of Medicine, Sendai, Japan

**Keywords:** NRF2, KEAP1, Cisplatin, Mitomycin C, Head and neck squamous cell carcinoma

## Abstract

Cisplatin (CDDP) resistance remains a major challenge in the treatment of recurrent head and neck squamous cell carcinoma (HNSCC). The KEAP1-NRF2 system, a central regulator of cellular redox homeostasis, is frequently altered in cancer, but its contribution to acquired CDDP resistance in HNSCC remains to be clarified. To address this, we investigated NRF2 activation in CDDP resistance using seven parental (P) HNSCC cell lines and their CDDP-resistant (CR) derivatives. Among these P-CR pairs, three CR lines exhibited elevated NRF2 expression; two harbored *KEAP1* mutations, and one had an *NFE2L2* mutation that were present in both P and CR lines. These NRF2-high CR lines showed upregulation of NRF2 target genes and enrichment of xenobiotic metabolism and reactive oxygen species pathways. Mitomycin C (MMC), a cytotoxic agent for its synthetic lethality in NRF2-activated cancer cells, demonstrated strong cytotoxicity specifically in these NRF2-high CR lines. Immunohistochemical analysis on clinical samples found that high NRF2 expression was significantly associated with poor prognosis and was frequently observed in recurrent tumors following chemoradiotherapy with CDDP. These results suggest that CDDP therapy, while initially effective, may paradoxically promote tumor progression and therapeutic resistance by aberrantly activating the KEAP1-NRF2 axis. This redox-driven adaptation highlights a critical characteristic in NRF2-hyperactivated HNSCC that is exploitable by MMC treatment.

## Highlights

• Cisplatin treatment induces NRF2 activation in HNSCC via KEAP1/NRF2 alterations.
• NRF2 activation promotes resistance to cisplatin in HNSCC.
• Mitomycin C shows cytotoxicity against NRF2-high, cisplatin resistant HNSCC cells.
• Clinical recurrent HNSCC tumors exhibit higher NRF2 levels than primary tumors.
• NRF2 activation by chemotherapy may drive redox-mediated malignant adaptation.

## Graphical abstract

**Figure:**
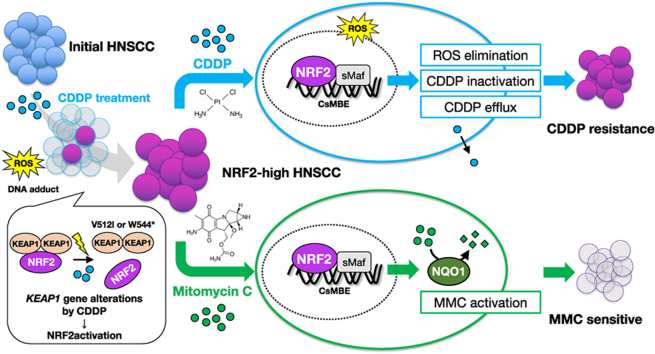

## 1 Introduction

Head and neck squamous cell carcinoma (HNSCC) arises from squamous epithelial cells in the head and neck regions, including the oral cavity, nasal sinuses, pharynx, larynx, salivary glands, and cervical esophagus. These tissues are vulnerable to exposure to external carcinogenic exposures, such as tobacco, alcohol, and viruses, which are known risk factors for HNSCC [1,2]. Over 60% of HNSCC patients present with locally advanced disease [3]. In addition to surgery, chemoradiotherapy is often used as a definitive treatment for locally advanced HNSCC, as it helps preserve essential head and neck functions. Radiotherapy or chemoradiotherapy is also employed postoperatively in high-risk cases, such as those with positive surgical margins or extranodal extension. Nevertheless, despite these definitive and postoperative therapies, treatment resistance and recurrence occur in 20–50% of cases [4]. Therefore, identifying new strategies to overcome treatment-resistant HNSCC is crucial for improving patient outcomes.

NRF2 (NF-E2-related factor 2; gene name *NFE2L2*) is a transcription factor regulating the cytoprotective responses by enhancing antioxidant defense and promoting xenobiotic detoxification [5,6]. KEAP1 (Kelch-like ECH-associated protein 1) acts as an adapter for the ubiquitin E3 ligase Cullin 3 (CUL3). KEAP1 forms a homodimer through its BTB domain [7]. Under steady-state conditions, the DLG and ETGE motifs in the Neh2 domain of NRF2 bind to the DC domain of KEAP1, resulting in the ubiquitination and subsequent degradation of NRF2 via the ubiquitin-proteasome system. Upon exposure to reactive oxygen species (ROS) or electrophiles, these stressors modify KEAP1, impairing its ability to bind NRF2. This leads to the stabilization and nuclear translocation of NRF2. In the nucleus, NRF2 forms a heterodimer with small Maf (sMaf) and binds to the CNC-sMaf binding element (CsMBE) sequence, thereby inducing the expression of target genes such as NAD(P)H: quinone oxidoreductase 1 (NQO1), glutathione synthetase, and drug efflux transporters [8,9]. Although NRF2 primarily functions in cytoprotecting through antioxidant defense and detoxification, it has also been implicated in squamous cell keratinization, carcinogenesis, and the suppression of proinflammatory cytokines [[10], [11], [12], [13]].

The KEAP1-NRF2 system has recently garnered significant attention in cancer research. In some cancer cells, NRF2 is constitutively activated, primarily due to somatic mutations that lead to loss of KEAP1 function and gain of NRF2 function, resulting in resistance to anticancer drugs and radiation [14]. Mutations in *NFE2L2* have been identified in approximately 10–36% of esophageal squamous cell carcinomas [15,16]. Additionally, *KEAP1* mutations are found in about 10–20% of lung adenocarcinomas, both of which are associated with poor prognosis [15,17,18]. In HNSCC, mutations in *KEAP1*, *NFE2L2* or *CUL3* are observed in approximately 22% of cases [19]. These genetic alterations in the KEAP1-NRF2 system are implicated in treatment resistance and poor prognosis in HNSCC.

Cisplatin (*cis*-diamminedichloroplatinum (II); CDDP) is a platinum-based chemotherapy drug that is commonly used in combination with radiotherapy to treat HNSCC. Upon entering the cell via passive diffusion, CDDP undergoes activation through the replacement of its two chloride ligands with water ligands. The activated form binds to DNA in cancer cells, thereby inhibiting cell proliferation and inducing apoptosis to exert its anticancer effects [20]. In addition to apoptosis, CDDP is also known to induce the generation of reactive oxygen species (ROS), which disrupt various cellular functions [[21], [22], [23]]. ROS-dependent cytotoxicity caused by CDDP can trigger ferroptosis in cancer cells via lipid peroxidation [24,25]. Although CDDP is widely used to treat various types of cancer, the development of resistance remains a major clinical challenge [26]. One such mechanism is pre-target resistance, in which CDDP is inactivated by glutathione or other molecules before it can bind to DNA [[27], [28], [29], [30], [31]]. This mechanism has been implicated in HNSCC and lung squamous cell carcinoma [32]. As the KEAP1-NRF2 system regulates intracellular glutathione levels and the expression of ATP-binding cassette transporters, it may contribute to CDDP resistance. However, the underlying mechanisms and evidence for targeting this pathway therapeutically remain unclear [33,34].

Developing therapeutic strategies that specifically target NRF2-activated cancers with poor prognosis has emerged as a clinically important issue for improving patient outcomes. We recently identified mitomycin C (MMC) as an effective agent against NRF2-activated cancers [35]. MMC was first isolated in 1955 from *Streptomyces caespitosus*, a species of actinomycete, as a compound with anticancer properties [36]. The quinone ring in MMC is reduced by quinone reductases, such as NQO1, into an active form with cytotoxic effects [37]. MMC is particularly effective in NRF2-activated cancers, where NQO1 expression is upregulated due to elevated NRF2 activity.

Based on the above background, the KEAP1-NRF2 system is expected to be an important therapeutic target in the treatment of HNSCC, particularly in cases where CDDP-based treatment has failed. We hypothesized that chemotherapy may induce constitutive NRF2 activation in HNSCC cells. In this study, using seven pairs of HNSCC cell lines, each consisting of parental cells and CDDP-resistant cells derived through prolonged CDDP-treatment, we discovered *KEAP1* mutations that drive constitutive NRF2 activation in two of the resistant lines. We demonstrated that MMC is specifically effective against CDDP-resistant HNSCC cell lines with NRF2 activation. Furthermore, we found that higher NRF2 expression levels in primary tumors are associated with poor prognosis and resistance to chemoradiotherapy in HNSCC patients.

## 2 Materials and methods

### 2.1 Reagents

CDDP (Fujifilm Wako, Osaka, Japan) was diluted in saline and stored at room temperature. MMC (Fujifilm Wako), Erastin (Tokyo Chemical Industry, Tokyo, Japan), deferoxamine (DFO; Tokyo Chemical Industry) and ferrostatin-1 (Fer-1; Sigma-Aldrich, Sent Louis, USA) were diluted in dimethyl sulfoxide (DMSO) and stored at −80°C. *N*-acetyl-l-cysteine (NAC; Sigma-Aldrich) was dissolved in water immediately before use. All compounds were further diluted in cell culture medium prior to application in experiments.

### 2.2 Parental (P) cell lines and CDDP-resistant (CR) derivatives cell lines

All parental (P) cell lines and their corresponding CDDP-resistant derivatives (CR) were obtained from the Department of Investigative Pathology, Tohoku university. CR cell lines have been already shown to acquire CDDP resistance compared to the P lines [38]. Among the ten available P-CR pairs, we used seven lines for this study (Table 1). The remaining three pairs were excluded due to technical limitations that made them unsuitable for this study. All cell lines were cultured in RPMI-1640 medium supplemented with 10% fetal bovine serum and 1% penicillin-streptomycin.

**Table 1:** Parent (P) and CDDP-resistant (CR) cell lines derived from HNSCC cell lines used in the study.

#	HNSCC cell line	Tissue origin	IC_50_ of CDDP (μg/mL)	
			P	CR
1	RPMI2650	Nasal septum	4.5	22.0
2	HSC-2	Oral cavity	7.9	26.0
3	HSC-3	Tongue	7.8	13.0
6	HO-1-u-1	Floor of Mouth	50.0	89.0
7	Ca9-22	Gingiva	10.0	24.0
8	HSC-4	Tongue	11.0	25.0
10	SAS	Tongue	4.1	8.2

IC_50_ values were cited from reference 38.

### 2.3 Human samples

To investigate whether NRF2 expression in primary HNSCC tumors predicts recurrent prognosis using NRF2 IHC analysis, Formalin-fixed paraffin embedded (FFPE) specimens were obtained from 31 HNSCC patients who underwent surgical resection or tissue biopsy at the Department of Oto-Rhino-Laryngology-Head and Neck Surgery, Tohoku University Hospital (Miyagi, Japan). All patients had received postoperative radiotherapy or chemoradiotherapy with CDDP. The diagnosis of squamous cell carcinoma was confirmed by pathologist assessment. Written informed consent was obtained from all patients prior to tissue collection. The studies were conducted in accordance with the ethical principles of the Declaration of Helsinki.

To compare NRF2 expression between primary and recurrent HNSCC tumors following postoperative radiotherapy or chemoradiotherapy, a second cohort of 7 HNSCC patients was analyzed. Ethical approval and consent procedures for this cohort were identical to those described above.

### 2.4 Immunoblotting

Whole-cell lysates were prepared by lysing cultured cells in SDS buffer containing 0.25 M Tris-HCl (pH 6.8), 8% SDS, and 20% glycerol. Protein concentration was determined using a BCA protein assay kit (Pierce, Life Technologies, Carlsbad, CA, USA) with bovine serum albumin as the standard. Antibodies used in this study are listed in Table S1.

### 2.5 Quantitative reverse transcription PCR (qRT-PCR)

Total RNA was extracted from cultured cells using the RNeasy Plus Mini Kit (QIAGEN, Venlo, Limburg, Netherland), following the manufacturer's instructions. Complementary DNA (cDNA) was synthesized from total RNA using the ReverTra Ace qPCR Kit (TOYOBO, Osaka, Japan). Gene expression was quantified using the KAPA SYBR Fast qPCR Kit (Nippon Genetics, Tokyo, Japan) on the StepOnePlus Real-Time PCR System (Thermo Fisher Scientific, Waltham, MA, USA). Expression data were normalized to *GAPDH*, and relative expression levels were calculated using the relative standard curve method. Primer sequences used for qRT-PCR are listed in Table S2.

### 2.6 NQO1-ARE-Luc promoter assay

The pNQO1-ARE-Luc reporter construct was previously described [39]. The pRL-tk plasmid, which expresses Renilla luciferase under the control of the thymidine kinase (TK) promoter, was used as an internal control. Cells were seeded into 6-well plates and cultured to approximately 70% confluency. The pNQO1-ARE-Luc plasmid and the pRL-tk plasmid were linearized and co-transfected into the cells using Viafect Transfection Reagent (Promega, Madison, Wi, USA). After 24 h of incubation, cell lysates were collected and transferred to white 96-well plates. Luciferase activity was measured using the Dual-Luciferase Reporter Assay System (Promega) with a Centro LB960 Luminometer (Berthold, Bad Wildbad, Baden-Wurttemberg, Germany). NQO1-ARE and pRL-tk activities were quantified by measuring firefly and Renilla luminescence. Reporter activity for each cell line was calculated as the ratio of firefly to Renilla luminescence.

### 2.7 Multiplex PCR for *KEAP1* and *NFE2L2* genes

Genomic DNA was extracted from cells using the DNeasy Blood and Tissue Kit (QIAGEN), following the manufacturer's instructions. DNA concentration was measured using a NanoPhotometer NP80 (Implen, Munich, Germany). Multiplex PCR was performed to amplify 28 amplicons covering the *NRF2* promoter region and the coding sequences of *KEAP1* and *NFE2L2*. Primer sequences are listed in Tables S3 and S4. The PCR protocol consisted of an initial denaturation at 95°C for 2 min, followed by 35 cycles of 98°C for 20 s, 60°C for 15 s, and 72°C for 30 s, with a final extension at 72°C for 2 min. PCR products were purified using the Gel/PCR DNA Isolation System (VIOGENE). Barcodes for the Illumina MiSeq sequencing were added to the purified PCR products via a second PCR, which consisted of 95°C for 2 min, followed by 20 cycles of 98°C for 20 s, 60°C for 15 s, and 72°C for 15 s.

### 2.8 Short-read amplicon sequencing

The concentration of barcoded PCR amplicons was assessed by peak molarity using D1000 ScreenTape and Sample Buffer (5067–5582 and 5067–5602, Agilent Technologies) on a TapeStation 2200 system (Agilent Technologies, Santa Clara, CA, USA). The pooled amplicons were then mixed, denatured, and clustered at a final concentration of 8 pM on a MiSeq system using the MiSeq Reagent Nano Kit V2 (Illumina, Santiego, CA, USA). Raw sequencing data were processed using the software provided with the MiSeq system. Gene mutations were identified by visualizing VCF files with Integrative Genomics Viewer (IGV) version 2.12.2.

### 2.9 Sanger sequencing analysis

The concentration of genomic DNA extracted for multiplex PCR was adjusted to 25 ng/μL. The first-round PCR was performed for trimming purposes, using a program consisting of an initial denaturation at 95°C for 3 min, followed by 30 cycles of 98°C for 20 s and 72°C for 30 s, with a final extension at 72°C for 2 min. PCR products were purified using ExoSAP-IT (Thermo Fisher Scientific). The purified PCR products were then sequenced using BigDye Terminator v3.1 Cycle Sequencing Kit and ABI 3500xL Genetic Analyzer (Thermo Fisher Scientific), following the manufacturer's instructions. Primer sequences are listed in Table S5.

### 2.10 RNA-seq analysis

Total RNA extracted for qRT-PCR analyses was quantified using a NanoPhotometer NP80 (Implen). RNA quality was assessed based on the RNA Integrity Number (RIN) and DV_200_ (percentage of RNA fragments longer than 200 nucleotides) using RNA ScreenTape and Reagents (5067–5576 and 5067–5577, Agilent Technologies) on a TapeStation 2200 system (Agilent Technologies). Ribosomal RNA (rRNA) was depleted using the MGIEasy rRNA Depletion Kit (MGI, Shenzhen, China). Double-stranded DNA (dsDNA) libraries were constructed from the rRNA-depleted eluates using the MGIEasy RNA Directional Library Prep Kit (MGI). Sequencing was performed on the DNBSEQ-G400RS (MGI) platform using the DNBSEQ-G400RS High-Throughput Sequencing Kit v1.0 (MGI) to obtain 150 bp paired-end reads.

Raw sequencing reads were processed using the Fastp, and transcript abundance was quantified using Salmon quant with the GRCh38 reference genome. For visualization purposes, such as heatmaps, expression data were normalized using trimmed mean of M-values (TMM) method with edgeR. For differential gene expression analysis, normalization and statistical testing data were performed using DESeq2. To detect *NQO1* single nucleotide variants (SNVs), RNA-seq reads were aligned to the reference genome using STAR and the results were visualized with IGV.

### 2.11 Pathway enrichment analysis

Gene set enrichment analysis (GSEA) was performed using GSEA software version 4.3.3. To validate NRF2 activation in cell lines, gene sets related to the KEAP1-NRF2 pathway from Reactome and the NRF2 pathway from WikiPathways were used. Hallmark gene sets were employed for both GSEA and single-sample GSEA (ssGSEA) to comprehensively characterize all cell lines. ssGSEA was conducted using the GenePattern software package [40]. Principal component analysis (PCA) was then performed based on the ssGSEA score.

### 2.12 Cell viability assay

Cells were seeded into clear 96-well plates at a density of 1 × 10^4^ cells per well for CDDP treatment and at 2 × 10^3^ cells per well for MMC treatment. After 16-h incubation, the culture medium was replaced with fresh medium containing the respective compounds. Cells were then incubated for 48 h with CDDP or 96 h with MMC. Following incubation, cell viability was assessed using the CellTiter 96 AQueous One Solution Cell Proliferation Assay (Promega) based on the MTS assay. After 1–2 h of incubation with the MTS reagent, absorbance at 492 nm, corresponding to the formazan product, was measured using an Absorbance 96 plate reader (ENZ–INS–A96, Enzo Life Sciences, Farmingdale, NY, USA).

### 2.13 CellROX green assay for ROS detection

Cells were seeded into black 96-well plates with clear bottoms at a density of 1 × 10^4^ cells per well. After 16-h incubation, the culture medium was replaced with fresh medium containing the designated compounds. Following 30 min, the medium was removed, and the cells were washed with PBS. CellROX Green reagent (Thermo Fisher Scientific) was then added to the wells. After an additional 30-min incubation, fluorescence intensity was measured using a PHERAstar FS microplate reader (BMG Labtech, Ortenberg, Germany).

### 2.14 Immunohistochemical (IHC) analysis

FFPE tissue sections were rehydrated and subjected to antigen retrieval by autoclaving in 10 mM sodium citrate buffer (pH 6.0). After treatment with 3% hydrogen peroxide, the sections were blocked using Protein Block Serum-Free (X0909, Dako, Santa Clara, CA, USA) and incubated sequentially with an anti-NRF2 antibody (sc-365949, Santa Cruz Biotechnology, TX, USA) for 16 h at 4°C, followed by incubation with the appropriate secondary antibody. NRF2 expression was evaluated under a 400x field of view. NRF2 IHC scores were determined based on the method developed by Kawasaki *et al*. [41]. The cutoff value for IHC score was determined using receiver operating characteristic (ROC) curve analysis with JMP Pro (SAS Institute, Cary, NC, USA).

### 2.15 Public databases

The Cancer Genome Atlas Program (TCGA) and Center for Cancer Genomics and Advanced Therapeutics (C-CAT) databases were used for mutation analysis. TCGA has been developed by the National Cancer Institute and the National Human Genome Research Institute since 2006 in the USA. It includes tumor samples from 530 HNSCC patients, collected prior to therapy, and analyzed by multi-omics approaches such as whole-genome and whole-exome sequencing. C-CAT database has been developed by the National Cancer Center Japan since 2018. The C-CAT database consists of targeted sequencing data from five cancer genome profiling (CGP) tests, FoundationOne CDx, FoundationOne Liquid CDx (Chugai Phamaceutical), GenMineTOP (Konika Minolta), Guardant360 CDx (Guardant Health Japan), and NCC Oncopanel (Sysmex Corporation). It includes tumor samples from 820 HNSCC patients who had undergone standard therapies for locally advanced or metastatic disease. TCGA data were downloaded using TCGAbiolinks in R (The R Foundation, Vienna, Austria) [42], and C-CAT data were accessed via the C-CAT portal. Mutations in *KEAP1* and *NFE2L2* genes were visualized using the G3viz package in R [43], except for data derived from Guardant360 CDx, which does not include *KEAP1* in its gene panel.

### 2.16 Mutational signature analysis for public databases

Mutational signatures analysis was performed using the deconstructSigs package in R [44]. Single base substitution (SBS) reference data (version 3.3) were obtained from the Catalogue of Somatic Mutations in Cancer (COSMIC). We performed mutational signature analysis using a combined set of signatures associated with platinum-based drug treatment and HNSCC In HNSCC, four major SBSs have been reported [45]: SBS1 (deamination of 5-methylcytosine), SBS2 and SBS13 (APOBEC enzyme activity), and SBS5 (possibly associated with aging, smoking and nucleotide excision repair deficiency). In addition, ten minor SBSs have been identified, including SBS3, SBS4, SBS7a, SBS7b, SBS7d, SBS16, SBS17a, SBS17b, SBS18, and SBS33. Two specific SBSs, SBS31 and SBS35, have been linked to platinum-based drug treatment [46]. As C-CAT is based on targeted panel sequencing, it includes only a subset of the genes analyzed in TCGA. Moreover, the set of target genes varies depending on the CGP test used. To ensure consistency, mutational signature analysis for both TCGA and C-CAT datasets was restricted to the 324 genes targeted by FoundationOne CDx, FoundationOne Liquid CDx panels.

### 2.17 Statistical analysis

For comparison of dose-response curves in Fig. 5A and B, an Extra sum-of-squares F test was conducted using Prism 10 (GraphPad Software, Santiego, CA, USA). A chi-squared test for Fig. 5B, Wilcoxon signed-rank test for Fig. 6C and F, and log-rank test for Fig. 6D and E were performed using JMP Pro. For all other statistical analysis, Student's *t*-test was performed using Prism 10. A *p*-value <0.05 was considered statistically significant.

## 3 Results

### 3.1 Increased NRF2 activation contributes to the resistance to CDDP

**Fig. 1:**
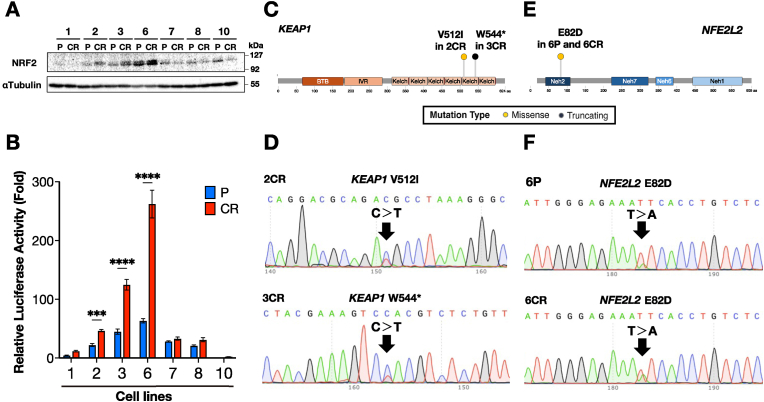
NRF2 activity and mutational analysis of *KEAP1* and *NFE2L2* genes in seven pairs of parental (P) and CDDP-resistant (CR) HNSCC cell lines. **A** Immunoblot analysis of NRF2 protein levels. Whole cell lysates were used, and α-tubulin served as a loading control. **B** Fold change of NQO1-ARE luciferase reporter activity. in cell lines. Values are normalized to the 10P cell line (set to 1). Quantitative data were expressed as the mean with SEM error bar of three independent experiments. ∗∗∗*P* < 0.001, ∗∗∗∗*P* < 0.0001. **C** and **E**, Lollipop plots showing genetic variants in *KEAP1* (**C**) and *NFE2L2* (**E**) identified by amplicon sequencing. **D** and **F**, Sanger sequencing profiles of mutations in *KEAP1* (**D**) and *NFE2L2* (**F**). In (**D**), sequencing chromatograms for 2CR (top) and 3CR (bottom) are shown. In (**F**), *NFE2L2* mutation profiles for 6P (top) and 6CR (bottom) are shown.

To investigate the relationship between NRF2 activation and CDDP resistance, we have used seven pairs of HNSCC cell lines, each consisting of a parental (P) cell lines and its CDDP-resistant (CR) derivatives. We assessed NRF2 activity and somatic mutations in the *KEAP1* and *NFE2L2* genes in these pairs of cell lines. To evaluate NRF2 protein expression levels in the seven cell line pairs, we performed immunoblotting analysis. Among the P lines, 6P exhibited markedly higher NRF2 expression compared to the other P lines (Fig. 1A). 6CR showed an even greater level of NRF2 protein expression than 6P. Similarly, 2CR and 3CR displayed increased NRF2 expression relative to their respective parental counterparts. In contrast, the remaining four CR lines did not show elevated NRF2 protein levels compared to the corresponding P lines.

We then assessed transcriptional activity of NRF2 by means of an NQO1-ARE luciferase reporter assay. Compared with their respective P lines (2P, 3P, and 6P), the CR lines 2CR, 3CR and 6CR showed significantly increased reporter activity (Fig. 1B). Among them, 6CR exhibited the highest induction of NQO1-ARE-driven luciferase activity. In contrast, the remaining four CR lines did not show substantial increases in reporter activity. These results support our hypothesis that NRF2 contributed to the acquisition of CDDP resistance in 2CR, 3CR, and 6CR.

We next investigated the molecular basis of NRF2 activation in the three CR lines. One plausible explanation is that somatic mutations in either the *KEAP1* or *NFE2L2* genes are responsible for elevated NRF2 activity. To examine this possibility, we designed amplicon-based short-read sequencing that targets both *KEAP1* and *NFE2L2,* and performed the analysis on all seven pairs of cell lines. As a result, we identified novel *KEAP1* mutations in two CR lines, 2CR and 3CR (Fig. 1C). Specifically, the V512I mutation found in 2CR and the W544∗ mutation found in 3CR were both located in the fifth Kelch domain of *KEAP1*. These mutations were not present in the corresponding P lines, suggesting that they arose during the acquisition of CDDP resistance in the CR lines. These *KEAP1* mutations identified in 2CR and 3CR were confirmed to be heterozygous by Sanger sequencing (Fig. 1D).

As noted earlier, 6P exhibited the highest basal NRF2 activity among all P lines and 6CR showed even greater NRF2 activity, as measured by the luciferase reporter assay. Amplicon-based sequencing of both *KEAP1* and *NFE2L2* revealed that both 6P and 6CR harbored a shared somatic mutation in *NFE2L2*, resulting in glutamic acid to aspartic acid substitution at residue 82 (E82D) within the ETGE motif (Fig. 1E). The E82D mutation has been previously reported in multiple squamous cell carcinomas and is associated with elevated NRF2 expression [47]. Thus, we identified three somatic mutations in the KEAP1-NRF2 pathway among all cell lines: two in *KEAP1* (2CR and 3CR) and one in *NFE2L2* (6P and 6CR). These mutations appear to underlie the marked increase in NRF2 activity observed in 2CR, 3CR, and 6CR.

Although 6P and 6CR harbor the same *NFE2L2* E82D mutation, the NQO1 reporter activity was substantially higher in 6CR compared to 6P. To explain this elevation in NRF2 activity, we consider several plausible explanations. One is that the E82D mutation exists in a heterozygous state in 6P but has become homozygous in 6CR. Similarly, certain loss-of-heterozygosity may explain observed phenomenon. To address this possibility, we examined Sanger sequencing chromatograms of the *NFE2L2* gene in both 6P and 6CR. The results clearly indicated that the E82D mutation remained heterozygous in both lines (Fig. 1F). Also, we could not find additional somatic mutations. These results ruled out a transition to homozygotic *NFE2L2* mutation in 6CR. Alternative possibility is that prolonged exposure to CDDP may have activated additional regulatory pathways that further enhance NRF2 activity.

### 3.2 CR cell lines with NRF2 activation show enrichment of NRF2 target gene expression

**Fig. 2:**
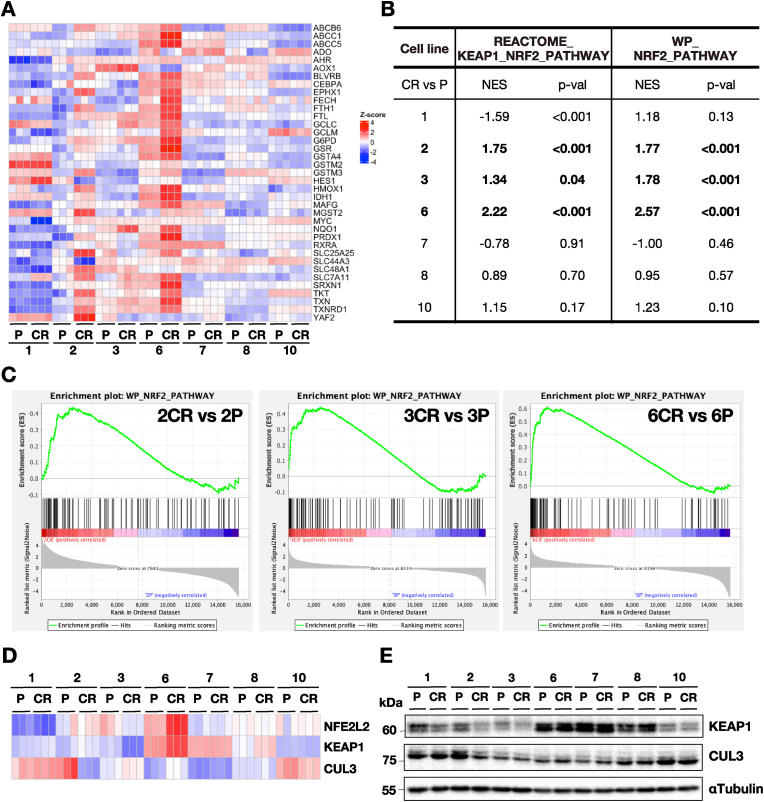
NRF2-related gene expression in seven pairs of parental (P) and CDDP-resistant (CR) HNSCC cell lines based on RNA-seq analysis. **A** Heatmap of representative NRF2 target gene expression levels from RNA-seq data. Notably, NRF2 target gene expression was elevated in the CR lines of the 2P-2CR, 3P-3CR, and 6P-6CR pairs compared to corresponding P lines. **B** Normalized enrichment score (NES) from GSEA of the NRF2 pathway using Reactome and WikiPathways (WP) gene sets. **C** Visualization of the NRF2 pathway from WikiPathways enriched by GSEA analysis. **D** Heatmap of *NFE2L2*, *KEAP1* and *CUL3* mRNA expression from RNA-seq analysis. **E** Immunoblot analysis of KEAP1 and CUL3 protein levels. Whole cell lysates were used, and α-tubulin served as a loading control.

To examine whether NRF2 target genes were upregulated in 2CR, 3CR, and 6CR cell lines, we performed RNA-seq analyses on all seven pairs of P and CR lines. The analysis provided a comprehensive overview of gene expression profiles across the cell lines. Based on a previous report [48], we extracted a set of genes which expressions are known to be enhanced by NRF2 (Fig. 2A). In fact, expressions of these NRF2 target genes were elevated in 2CR, 3CR, and 6CR compared to their respective P lines. In contrast, while some moderate variations were observed, the remaining four cell line pairs did not show consistent increase of this set of NRF2 target gene expression between P and CR lines. Another notable observation was that the expression of NRF2 target genes varied among cell line pairs, corresponding with NRF2 protein expression levels and activities (see Fig. 1A and B).

To validate NRF2 activation in cell lines, we then performed GSEA using gene sets related to the KEAP1-NRF2 pathway from Reactome and the NRF2 pathway from WikiPathways. To our expectation, enrichment of the KEAP1-NRF2 pathway and activation of NRF2 target genes expressions were significant in 2CR, 3CR and 6CR (Fig. 2B and C). These results indicate that among the seven CR lines, 2CR and 3CR acquired NRF2 activation through somatic mutations in *KEAP1* during prolonged exposure to CDDP. Similarly, 6CR, which already harbored the *NFE2L2* E82D mutation at the 6P stage, exhibited further NRF2 activation.

We also examined the mRNA expression levels of *NFE2L2*, *KEAP1* and *CUL3* in the seven pairs of HNSCC cell lines. Among them, 6CR exhibited the highest expressions of both *NFE2L2* and *KEAP1* (Fig. 2D). Moderate elevation of *KEAP1* was also observed in 7P, 7CR, and 8CR. In contrast, both 1P and 1CR showed the lowest levels of *NFE2L2* mRNA, along with relatively low *KEAP1* expression. In 2P, *CUL3* expression was markedly elevated, but it was reduced in 2CR. 3CR exhibited lower *KEAP1* expression than its parental counterpart. These results indicate that the expression levels of *NFE2L2*, *KEAP1* and *CUL3* mRNAs vary across HNSCC cell lines and are affected to some extent by the CDDP treatment. It should be noted that NRF2 protein levels are primarily regulated at the level of protein stability [49]. Therefore, the observed changes in *NFE2L2*, *KEAP1* and *CUL3* mRNA levels in these CDDP-treated HNSCC cell lines suggests that these three genes are also subject to complex regulations, particularly under conditions of cellular stress. The pronounced upregulation of both *NFE2L2* and *KEAP1* in 6CR suggests the presence of a unique regulatory mechanisms affecting the expression of these genes in this specific context.

To examine whether changes in *KEAP1* and *CUL3* mRNA levels affect the corresponding protein levels, we performed Western blotting analysis. We observed that KEAP1 protein expression was markedly high in 6P/6CR, 7P/7CR, and 8P/8CR, with comparable expression levels across these pairs (Fig. 2E). These protein levels reflected, although not perfectly, the changes observed at the mRNA level. In the case of 6P and 6CR, NRF2 harbors a mutation that enables the escape from KEAP1-mediated degradation, thereby maintaining high levels of NRF2 protein. In 2CR, KEAP1 protein expression was reduced compared to 2P, which may additionally underlie the moderate increase in NRF2 levels in this cell line. The mechanisms responsible for changes in KEAP1 protein levels remain unclear. We speculate that additional regulatory pathways affecting *KEAP1* mRNA expression may be activated under severe stress conditions, such as prolonged cell culture or exposure to CDDP.

CUL3 protein expression largely reflected its mRNA expression levels across the seven pairs of cell lines. High levels of CUL3 protein were observed in 1P/1CR, 2P, and 10P/10CR. When comparing P and CR lines, CUL3 protein expression in 2P and 2CR showed divergence, whereas the other cell line pairs exhibited comparable expression levels between P and CR. These findings suggest that while the basal levels of CUL3 protein vary among HNSCC cell lines, exposure to CDDP has minimal impact on CUL3 protein expression.

### 3.3 NRF2 activation in CR cell lines contributes to ROS elimination and enhanced xenobiotic metabolisms

**Fig. 3:**
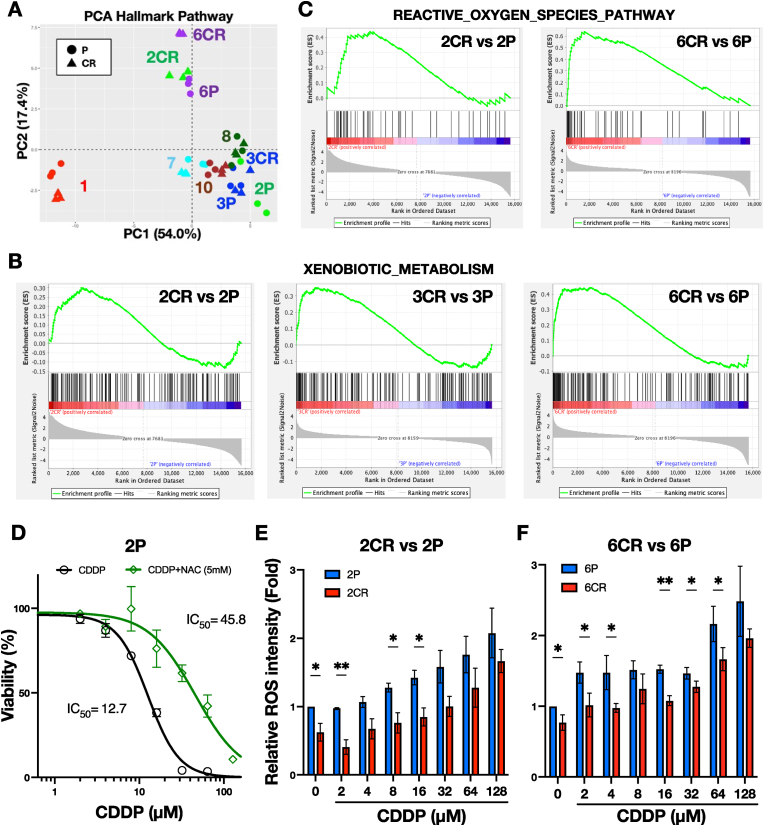
CDDP resistance via enhanced xenobiotic metabolism and ROS elimination in NRF2-activated CR cell lines. **A** Principal component analysis (PCA) of HNSCC cell lines based on ssGSEA scores from Hallmark pathways. **B** and **C,** Visualization of Hallmark xenobiotic metabolism pathway (**B**) and reactive oxygen species pathway (**C**) by GSEA. **D**, Dose-response curve of CDDP with or without NAC treatment in 2P. Data were expressed as the mean with SEM error bar of three independent experiments. **E** Relative ROS levels in 2P and 2CR, as measured by CellROX. Values are normalized to the untreated 2P (set to 1). **F**, ROS levels in 6P and 6CR, as measured by CellROX. Values are normalized to the untreated 6P (set to 1). Note the reduced ROS levels in 2CR and 6CR compared to their respective P cell lines. Quantitative data in (**E**) and (**F**) were expressed as the mean with SEM error bar of three independent experiments. ∗; *P* < 0.05, ∗∗; *P* < 0.01.

To investigate how CDDP treatment contributes to the phenotypic characteristics of CR cell lines through NRF2 activation, we applied ssGSEA using the Hallmark gene set to all cell lines and performed PCA based on the ssGSEA scores (Fig. 3A). An important feature of ssGSEA is that it provides an enrichment score for each gene set in individual samples [40]. If the principal components (PCs) differ between P and CR cell lines, gene sets contributing to these PCs may offer insight into the mechanism of CDDP resistance. PCA revealed that the four CR lines, 1CR, 7CR, 8CR, and 10CR, clustered closely with their respective parental counterparts. In contrast, 2CR and 3CR, both harboring somatic *KEAP1* mutations that lead to NRF2 activation, exhibited a shift along PC2. Similarly, 6CR, another NRF2-activated line, displayed a shift in the same direction.

We next performed GSEA using Hallmark gene sets to compare 2CR vs 2P, 3CR vs 3P, and 6CR vs 6P. Upregulated pathways for each comparison are listed in Tables S6, S7 and S8. Among these, xenobiotic metabolism was commonly and significantly enriched across all three CR lines (Fig. 3B). The reactive oxygen species pathway was the most enriched in 6CR and was also enriched in 2CR (Fig. 3C). Both of these pathways are well-established NRF2-regulated processes involving numerous NRF2 target genes. These findings further support the possibility that NRF2 plays a key role in mediating CDDP resistance in 2CR, 3CR, and 6CR.

We hypothesized that NRF2-mediated ROS elimination may contribute to CDDP resistance in CR lines. To test this hypothesis, we treated 2P with NAC, a ROS scavenger. We found that NAC rescued 2P from CDDP-induced cytotoxicity in a dose-dependent manner, with a threefold increase in IC_50_ values (Fig. 3D). We next selected 2CR and 6CR as representative NRF2-activated CR lines and assessed intracellular ROS accumulation using CellROX assay. In all examined cell lines, ROS levels increased with higher concentration of CDDP (Fig. 3E and F). Notably, both 2CR and 6CR exhibited lower ROS accumulation than their parental counterparts, 2P and 6P, under CDDP treatment. This reduction in ROS levels is associated with NRF2 activation, suggesting that NRF2 contributes to resistance to CDDP.

### 3.4 NRF2 activated CR cell lines acquire the resistance to ferroptosis

**Fig. 4:**
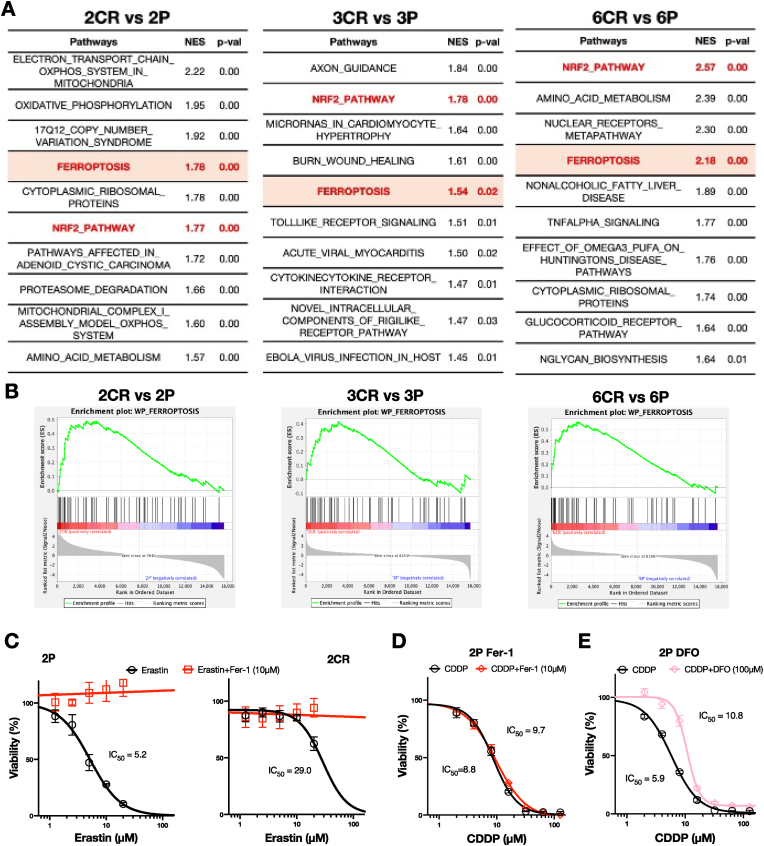
Enrichment of ferroptosis pathway in NRF2-high CR cell lines **A** Top 10 enriched pathways from WikiPathways (WP) gene set in NRF2-high CR cell lines compared to their respective P lines. The ferroptosis pathway is highlighted in red text with an orange background. The NRF2 pathway is indicated in red text. NES: normalized enrichment score. **B** Visualization of ferroptosis pathway by GSEA. **C** Dose-response curves of cytotoxicity induced by Erastin alone or in combination with ferrostatin-1 (Fer-1; 10 μM) in the 2P (left) and 2CR (right) cell lines. Data were expressed as the mean with SEM error bar of three independent experiments. **D** Dose-response curves for CDDP with Fer-1 (10 μM) in 2P. In 2P, the cytotoxicity of CDDP was not reduced by Fer-1. **E** Dose-response curves for CDDP with deferoxamine (DFO; 100 μM) in 2P. The cytotoxicity of CDDP was slightly reduced by DFO.

We further sought to identify common mechanisms of CDDP resistance in the three NRF2-activated CR cell lines by performing alternative pathway analysis using the WikiPathways database and identified ferroptosis among the top five commonly enriched pathways in 2CR, 3CR, and 6CR (Fig. 4A and B). To functionally validate this finding, we performed cytotoxicity assays in the presence or absence of Erastin, a ferroptosis inducer, using 2P and 2CR (Fig. 4C). Erastin induced cell death in both lines. However, 2CR showed approximately five-fold attenuation of cytotoxicity compared to 2P, suggesting that increased NRF2 activity contributes to the suppression of ferroptosis in 2CR. The cytotoxic effect of Erastin was almost completely reversed by the addition of Fer-1, a ferroptosis inhibitor, confirming that the observed cell death in both 2P and 2CR was ferroptosis-dependent.

**Fig. 5:**
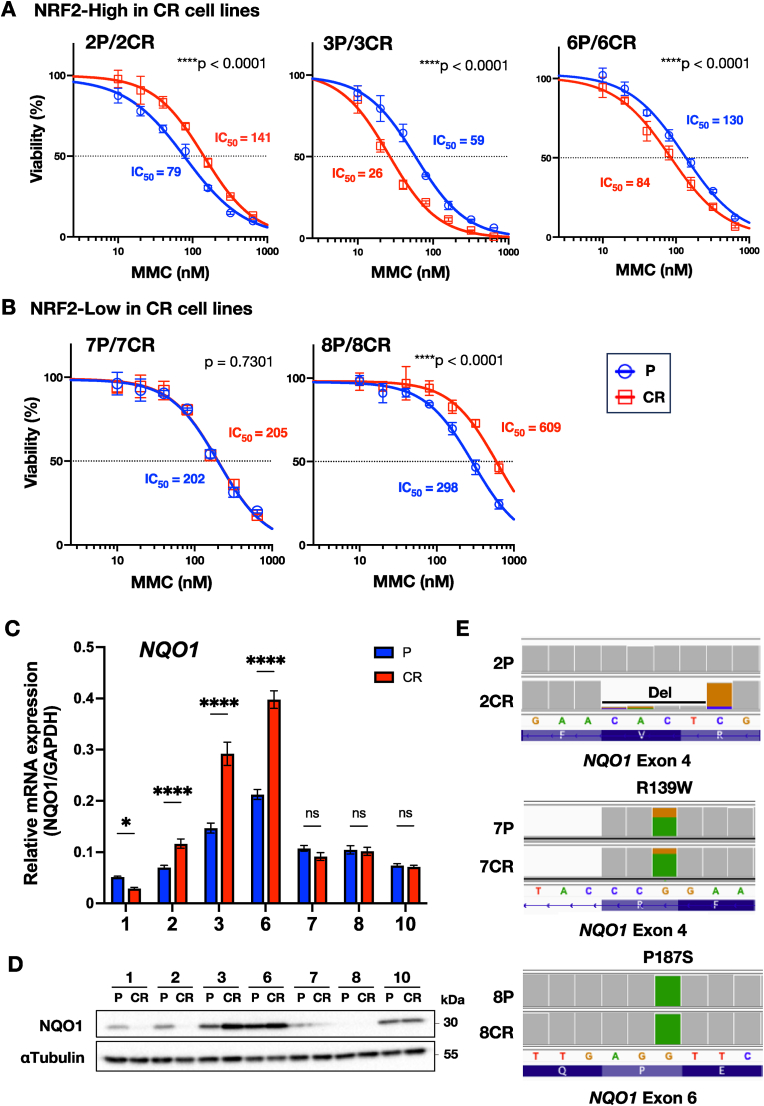
Efficacy of MMC in NRF2-activated, CDDP-resistant HNSCC cell lines. **A** Dose-response curves for MMC in NRF2-activated CR lines. Blue lines indicate P cell lines and red lines indicate CR cell lines. 2P/2CR (left), 3P/3CR (middle), 6P/6CR (right). Note the enhanced MMC sensitivity in 3CR and 6CR cell lines. **B** Dose-response curves for MMC in NRF2-non-activated CR cell lines. 7P/7CR (left), 8P/8CR (right). Data in (**A**) and (**B**) were expressed as the mean with SEM error bar of three independent experiments. ∗∗∗∗*P* < 0.0001. **C** Relative mRNA expression levels of *NQO1* determined by qRT-PCR. *GAPDH* was used as a housekeeping control. Quantitative data were expressed as the mean with SEM error bar of three independent experiments. ∗; *P* < 0.05, ∗∗∗∗*P* < 0.0001. **D** NQO1 protein levels analyzed by immunoblotting. α-tubulin served as a loading control. **E** Visualization of *NQO1* mutations from RNA-seq mapping data. Upper, middle and lower panels show 2P/2CR, 7P/7CR, and 8P/8CR, respectively.

**Fig. 6:**
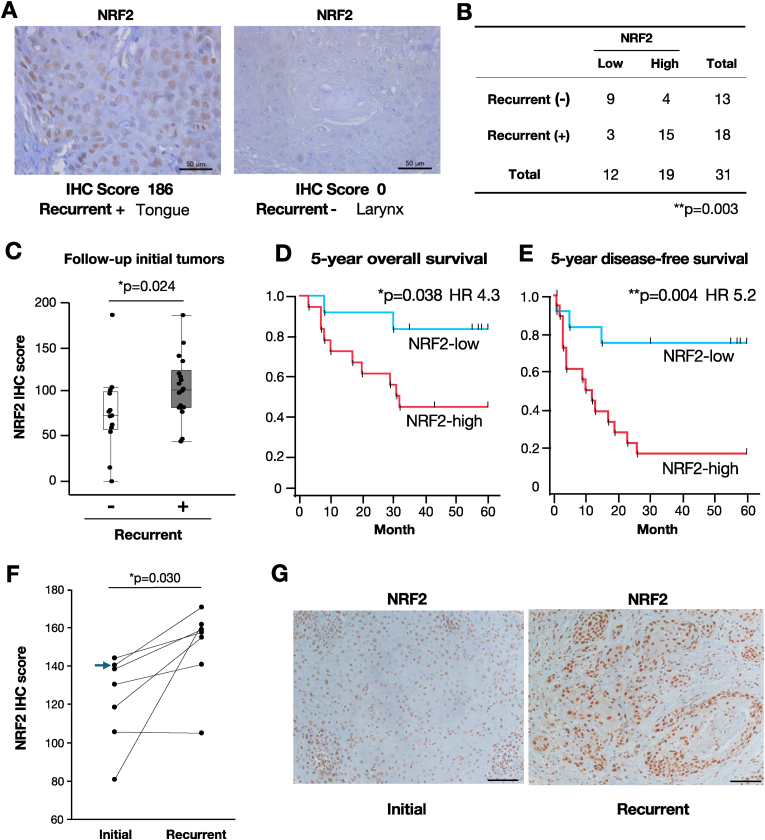
NRF2 expression and its association with recurrence in clinical HNSCC samples. **A** Representative IHC images of NRF2 in two patients. The right panel shows an initial tumor with an IHC score of 0 from a patient who remained recurrence-free. The left panel shows an initial tumor with an IHC score of 186 from a patient who experienced recurrence. The scale bar shows 50 μm. **B** Cross-tabulation of NRF2 IHC score and recurrence status. NRF2-high and NRF2-low groups were defined as IHC scores >82 and ≤ 82, respectively. Note that the NRF2-high group exhibited a higher frequency of recurrence. **C** Comparison of NRF2 IHC scores in initial HNSCC tumors between patients with and without recurrence. **D** and **E,** Kaplan-Meier curves of 5-year overall survival (**D**) and disease-free survival **(E**) stratified by NRF2 IHC status. The NRF2-high group showed significantly worse prognosis in both metrics. **F** NRF2 IHC scores in paired initial and recurrent tumor samples from seven patients. Each line connects the two samples from the same patient. The blue arrow indicates the case shown in panel (**G**). **G** Representative IHC images of NRF2 in paired tumor samples (initial, left; recurrent, right) from the same patient highlighted in (**F**). The scale bar shows 50 μm.

Since CDDP has been reported to induce cell death via ferroptosis, we investigated whether CDDP-induced cell death could be attenuated by ferroptosis inhibitors. Two ferroptosis inhibitors, Fer-1 and DFO, were administered in combination with CDDP to 2P cell lines. Ferrostatin-1 inhibits lipid peroxidation at a late stage of ferroptosis, while DFO is an iron chelator that reduces ROS production via the Fenton reaction. However, co-treatment with Fer-1 did not reduce CDDP-induced toxicity (Fig. 4D). On the other hand, co-treatment with DFO led to a mild reduction in CDDP-induced toxicity (Fig. 4E). These results suggest that, in the 2P cell line at least, CDDP induces cell death primarily through ROS-mediated mechanisms rather than ferroptosis.

### 3.5 MMC is effective against CR cell lines through NRF2 activation

Our findings indicated thus far highlight the importance of NRF2 activation in the development of novel therapeutic strategies for CDDP-resistant HNSCC. Based on this, we focused on MMC, a chemotherapeutic agent traditionally used in the treatment of anal and bladder cancers [50]. MMC has been identified as an effective agent against NRF2-activated cancers [35]. The quinone ring in MMC is reduced by quinone reductases, such as NQO1, into an active form with cytotoxic effects [37]. Since NQO1 expression is upregulated due to elevated NRF2 activity, MMC is expected to be particularly effective in NRF2-activated cancers.

Therefore, we next assessed the cytotoxic effects of MMC for NRF2-high CR, *i.e.,* 2CR, 3CR, and 6CR, and respective P cell lines. As shown in Fig. 5A, we found that MMC was significantly more effective for 3CR and 6CR, which exhibited high NRF2 activity, than their respective P lines, 3P and 6P. By contrast, 2CR did not show increased sensitivity to MMC compared to 2P. Similarly, MMC was less effective in NRF2-low 7P/7CR and 8P/8CR (Fig. 5B). In response to MMC, 7P and 7CR showed similar sensitivity, while 8CR was less responsive than 8P.

One plausible explanation for the low sensitivity of 2CR against MMC is that there may be strong inactivation of NQO1 in the 2CR cell line. *NQO1* mRNA levels were significantly elevated in 2CR, 3CR, and 6CR cell lines compared to their respective parental counterparts, while no significant changes were observed in the other cell line pairs (Fig. 5C). Consistently, NQO1 protein levels were also increased in 3CR and 6CR (Fig. 5D). In contrast, NQO1 protein expression was decreased in 2CR compared to that in 2P.

To investigate this discrepancy, we examined the RNA-seq-based genome mapping data of *NQO1*, since single nucleotide variants (SNVs) at codons R139W and P187S have been reported to cause loss of NQO1 protein expression [51]. These SNVs are located in exons 4 and 6 of the *NQO1* gene, respectively. Indeed, we found that 7P/7CR and 8P/8CR harbored a G > A substitution at R139W and P187S, respectively (Fig. 4E). By contrast, we identified a four-base deletion followed by a one-base substitution in exon 4 of *NQO1* gene in 2CR cell line. Despite the gene analysis, however, the cause of decreased NQO1 protein expression in 1CR cell line remains unclear. These findings thus demonstrate that protein-level assessment of NQO1 is essential for evaluating the potential efficiency of MMC treatment.

In summary, our results suggest that MMC may be a promising therapeutic agent for CDDP-resistant HNSCC cell lines with string NRF2 activation. MMC appears to exert its anti-tumor effects more selectively in cancer cells with high NQO1 expression, a downstream target of NRF2, such as NRF2-activated HNSCCs.

### 3.6 High NRF2 expression in clinical HNSCC sample is associated with recurrence and poor prognosis by NRF2 IHC scoring

We indicated that CDDP treatment induced NRF2 activation via *KEAP1* mutations through *in vitro* experiments. To explore whether this NRF2 activation also occurs in patients, we examined clinical HNSCC specimens to assess NRF2 expression after CDDP-based therapy. As an initial validation of NRF2 IHC as a marker of disease aggressiveness, we analyzed FFPE tumor samples from 31 patients with HNSCC who underwent surgical resection at Tohoku University Hospital. Clinical features of these 31 HNSCC patients were summarized in Table S9. The patients were categorized into two groups: 13 patients who were cured without recurrence and 18 patients who developed recurrence after surgery. All patients received postoperative radiotherapy or chemoradiotherapy with CDDP.

To further classify the specimens of the 31 HNSCC cases based on NRF2 expression levels, the samples were subdivided into two groups: NRF2-high and NRF2-low, according to IHC analysis. Representative IHC images are shown in Fig. 6A. The NRF2-negative case (IHC score = 0; right panel) was from the cured-case group, while the NRF2-positive case (IHC score = 186; left panel) was from the recurrent group. To define the threshold for classification, we calculated a cutoff value for the NRF2 IHC score using a ROC curve analysis (Fig. S1). Based on the ROC analysis using recurrence status and IHC scores, the cutoff was determined to be 82.

Using the cutoff score of 82, 9 patients (69%) in the cured group were classified as NRF2-low and 4 patients (31%) as NRF2-high. In stark contrast, among the recurrent cases 3 patients (17%) were classified as NRF2-low and 15 (83%) as NRF2-high (Fig. 6B). The NRF2 IHC scores were significantly higher in patients who experienced recurrence compared to those who remained recurrence-free (Fig. 6C). These findings indicate that NRF2-high expression is significantly more prevalent in recurrent cases, suggesting that NRF2 expression in primary tumors serves as a prognostic indicator in HNSCC.

We next compared five-year overall survival (OS) and disease-free survival (DFS) between the NRF2-high and NRF2-low groups. The OS curve showed that 56% of NRF2-high patients died within 32 months, compared to only 17% of NRF2-low patients (Fig. 6D). Similarly, the DFS curve showed that 83% of NRF2-high patients experienced relapse, whereas only 25% of NRF2-low patients experienced relapse (Fig. 6E). These results demonstrate significant differences in both OS and DFS between the NRF2-high and NRF2-low groups.

Then, to assess whether radiotherapy or chemoradiotherapy affects NRF2 expression following treatment, we compared NRF2 IHC scores in seven patients for whom both primary (initial) and recurrent tumor samples were available. In six out of seven cases, the NRF2 IHC scores were markedly higher in the recurrent tumors than those in the initial tumors, with a significant difference observed between the two groups (Fig. 6F). Representative NRF2 staining images of paired samples from the same patient are shown in Fig. 6G, revealing markedly elevated NRF2 expression in the recurrent tumor. All recurrent tumors in these patients had been treated with postoperative radiotherapy or chemoradiotherapy with CDDP. These results further support the notion that NRF2 expression is associated with tumor recurrence and/or resistance to therapy in HNSCC.

To investigate whether NRF2 activation observed in recurrent clinical HNSCC tumors was attributable to CDDP exposure, we performed mutational signature analysis using two datasets: The Cancer Genome Atlas (TCGA) [52], which predominantly contains treatment-naïve HNSCC tumors, and the Center for Cancer Genomics and Advanced Therapeutics (C-CAT) [53], which includes a higher proportion of recurrent HNSCC tumors following treatment. Although the overall mutational spectra were similar between the two datasets (Fig. S2A), a platinum-associated SBS signature, which is indicative of prior CDDP exposure, was detected exclusively in the C-CAT dataset (Fig. S2B).

Because the NRF2 activation appeared to be driven by *KEAP1* or *NFE2L2* gene alterations during CDDP treatment, we compared the frequency and distribution of these mutations between TCGA and C-CAT datasets. In the TCGA dataset, *KEAP1* mutations were detected in 20 cases (4%), and in the C-CAT dataset, in 28 cases (4%), with mutations distributed across various functional domains (Fig. S3A). However, when focusing on single nucleotide substitutions, mutations in the BTB and DC domains were more frequently observed in the C-CAT dataset (Fig. S3B). Notably, both *KEAP1* mutations identified in our CR lines were C > T single base substitutions, consistent not only with the overall SBS pattern observed in HNSCC but also with the *KEAP1*-specific SBS pattern (Fig. S3C). In parallel, and consistent with previous reports, *NFE2L2* mutations were detected in 30 cases (6%) in the TCGA dataset and 90 cases (11%) in the C-CAT dataset, with the majority clustering within the DLG and ETGE motifs, known hotspots for NRF2 activation [54] (Fig. S4). The higher frequency of *NFE2L2* mutations in the C-CAT dataset may be attributed to its enrichment for recurrent or poor-prognosis cases, which are more likely to have undergone prior CDDP-based therapy.

In summary, these results indicate that even in clinical HNSCC specimens, CDDP-containing therapy may induce mutations in functional domains, including the DC domain of *KEAP1*, causing elevated NRF2 IHC scores. This is consistent with the *in vitro* results (See Fig. 1C).

## 4 Discussion

This study focuses on the roles of NRF2 in conferring resistance to CDDP in HNSCC cells. To investigate the underlying mechanisms and functional consequences of NRF2 activation in CDDP-treated cells, we analyzed seven CDDP-resistant (CR) cell lines alongside their parental (P) counterparts. Among them, three CR cell lines, 2CR, 3CR, and 6CR, exhibited elevated NRF2 activity following prolonged exposure to CDDP. Of these, 2CR and 3CR harbored somatic C > T single base substitutions in the DC domain of *KEAP1* gene that were absent in the respective P lines. In contrast, both 6P and 6CR carried a mutation in the *NFE2L2* gene, and both exhibited elevated *NFE2L2* mRNA expression, with 6CR showing a substantially higher increase at both mRNA and protein levels. These findings support the notion that enhanced NRF2 activation may contribute to CDDP resistance in these CR cell lines by upregulating genes involved in drug metabolism and ROS elimination.

In this study, we provided evidence that MMC is a promising anti-cancer agent for CDDP-resistant, NRF2-high HNSCC cells. Moreover, IHC analyses of clinical specimens revealed that recurrent tumors, which had undergone postoperative chemoradiotherapy including CDDP, displayed significantly higher NRF2 expression compared to their matched primary tumors. These findings suggest that CDDP treatment frequently leads to increased NRF2 expression.

We identified two CR lines, 2CR and 3CR, both of which acquired somatic mutations in the DC domain of *KEAP1* during the establishment of CDDP resistance. In both CR lines, the *KEAP1* mutations led to NRF2 activation, suggesting the KEAP1-NRF2 axis as a key molecular mechanism underlying CDDP resistance. The KEAP1-NRF2 system primarily functions to eliminate ROS and xenobiotic compounds, including therapeutic agents. Accordingly, the enrichment of gene sets related to xenobiotic metabolism and ROS pathways in these CR lines likely reflects the establishment of NRF2-high phenotype. Consistent with these observations, we found reduced ROS accumulation upon CDDP treatment in NRF2-high CR lines, suggesting that enhanced ROS elimination contributes significantly to CDDP resistance in these cells. Importantly, these findings raise the concern that CDDP exposure, while intended to eradicate tumor cells, may inadvertently promote malignant progression in HNSCC by inducing somatic *KEAP1* mutations and subsequent NRF2 activation.

To target NRF2 therapeutically, we proposed the use of MMC as a synthetic lethal strategy for treating CDDP-resistant, NRF2-high HNSCC. The NRF2-high CR lines lacking *NQO1* SNVs, specifically 3CR and 6CR, exhibited marked sensitivity to MMC, suggesting that the anticancer effect of MMC is highly dependent on functional NQO1 expression. Activations of NRF2-related pathways have been shown to enhance cancer cell survival and malignancy by enabling better tolerance to oxidative and other stresses [55]. Among NRF2 target genes, NQO1 plays a critical role in detoxifying oxidative and xenobiotic stress. In animal models, NQO1 is commonly used as a reliable readout of NRF2 activity [9]. However, in human cancers, several SNVs in the *NQO1* gene have been identified that impair its function through mechanisms such as protein instability [56,57]. As previously reported, approximately 18.1% of the Japanese population carries SNVs of *NQO1*, such as R139W and P187S, which result in NQO1 deficiency [51]. These mutations destabilize the NQO1 protein, promoting its degradation via the proteasome system [58]. In this study, we detected *NQO1* mutations at R139W in 7P/7CR and P187S in 8P/8CR, both of which showed resistance to MMC. Additionally, we identified a four-base deletion followed by a one-base substitution in the *NQO1* gene in 2CR, which was not present in 2P, suggesting that this mutation may have arisen during CDDP treatment. The resulting NQO1 deficiency likely contributed to MMC resistance in 2CR. These findings underscore the potential emergence of MMC resistance due to somatic *NQO1* mutations during CDDP-based therapy. Therefore, in the context of recurrent HNSCC, it is important to consider not only NRF2 activation but also genetic alterations in *NQO1* when selecting therapeutic strategies.

In our clinical sample analyses using the NRF2 IHC scoring, NRF2-high HNSCC cases appeared to be strongly associated with tumor recurrence and showed significantly worse outcomes in both OS and DFS compared to those of NRF2-low cases. Although IHC analysis is a simple and widely used method in clinical practice for predicting prognosis and treatment response, to our knowledge, no prior studies have linked NRF2 expression levels to patient prognosis based on the IHC analysis in HNSCC. We propose that NRF2 IHC scoring should be implemented in routine clinical practice to guide personalized treatment strategies for patients with NRF2-activated HNSCC. Importantly, NRF2 IHC scores in recurrent tumors, particularly those treated with postoperative radiotherapy or chemoradiotherapy, were significantly higher than in corresponding initial tumors. This observation suggests that treatment-induced mutations, especially in the functional domains of *KEAP1*, may contribute to NRF2 activation, consistent with our comparative analysis of TCGA and C-CAT datasets. Based on these findings, we conclude that NRF2 IHC scoring is a valuable tool for predicting the prognosis of HNSCC. Nonetheless, it is important to note that the sensitivity and specificity of NRF2 IHC scoring were 83.3% and 69.2%, respectively. Further evaluations in larger cohorts of HNSCC patients will be essential to establish its clinical utility.

In this study, we demonstrated that NRF2 activation, driven by genetic alterations in the KEAP1–NRF2 system induced by CDDP, underlies CDDP resistance in HNSCC. Moreover, we showed that MMC exhibits selective efficacy against NRF2-activated, CDDP-resistant HNSCC cell lines. Importantly, our findings highlight a paradox in current therapeutic practice: CDDP, a standard-of-care chemotherapeutic agent, can inadvertently promote NRF2 activation, thereby contributing to resistance. Based on these insights, we propose a novel treatment strategy for recurrent HNSCC that includes assessing genetic alterations in *KEAP1*, *NFE2L2*, and *NQO1* to guide the potential use of MMC. Although MMC is approved for the treatment of HNSCC, it is not widely utilized and remains outside the current standard of care. Therefore, clinical studies evaluating MMC in NRF2-activated, CDDP-resistant HNSCC are warranted and may provide a path toward biomarker-driven, personalized therapy. We believe that our findings offer a foundation for redox-guided therapeutic stratification and may ultimately improve outcomes in patients with recurrent HNSCC.

## CRediT authorship contribution statement

**Yuki Nakayama:** Conceptualization, Data curation, Formal analysis, Investigation, Methodology, Software, Validation, Visualization, Writing – original draft, Writing – review & editing. **Keiko Taguchi:** Conceptualization, Project administration, Supervision, Writing – review & editing. **Shun Wakamori:** Conceptualization, Formal analysis, Investigation, Methodology, Resources. **Akira Uruno:** Methodology. **Akihito Otsuki:** Methodology, Software, Supervision. **Akira Ohkoshi:** Resources, Supervision. **Hidekazu Shirota:** Data curation, Resources, Writing – review & editing. **Tomoyuki Iwasaki:** Data curation, Resources. **Yukio Katori:** Resources, Supervision. **Masayuki Yamamoto:** Funding acquisition, Project administration, Supervision, Writing – review & editing.

## Ethics declarations

Written informed consent was obtained from all patients prior to tissue collection. The studies were conducted in accordance with the ethical principles of the Declaration of Helsinki.

## Financial Support

This research was supported by JSPS KAKENHI 19H05649 (to M.Y.) and the Advanced Research Center for Innovations in Next-Generation Medicine (INGEM) at Tohoku University.

## Declaration of competing interest

The authors declare the following financial interests/personal relationships which may be considered as potential competing interests: Masayuki Yamamoto reports financial support was provided by Japan Society for the Promotion of Science. Masayuki Yamamoto reports financial support was provided by The Advanced Research Center for Innovations in Next-Generation Medicine (INGEM) at Tohoku University. If there are other authors, they declare that they have no known competing financial interests or personal relationships that could have appeared to influence the work reported in this paper.

## Data Availability

The RNA-seq data generated in this study have been deposited in the Gene Expression Omnibus (GEO) under accession number GSE293361.
