# Supplementary material for: Cisplatin-induced genetic alterations in KEAP1 promote therapeutic resistance in head and neck squamous cell carcinoma
Source: Redox Biol. 2025 Aug 11;86:103819. doi: 10.1016/j.redox.2025.103819 (PMC12540038; doi:10.1016/j.redox.2025.103819)
Supplement: Multimedia component 1 [file mmc1.pdf]

Supplementary Information

Supplementary Table S1. Antibodies used for immunoblotting

Antibody	Catalog number	Company	Dilution
Anti-αTubulin	T9026	Sigma-Aldrich	1:500
Anti-NQO1	Ab2346	Abcam	1:1000
Anti-NRF2	#103	Maruyama et al. [1]	1:200
Anti-KEAP1	#144	Watai et al. [2]	1:100
Anti-CUL3	611848	BD Biosciences	1:1000

Supplementary Table S2. Primers used for qRT-PCR

Human gene		5'-3' sequence
NQO1	F	GTCATTCTCTGGCCAATTCAGAGT
	R	TTCCAGGATTTGAATTCGGG
GAPDH	F	GAAGGTGAAGGTCGGAGTC
	R	GAAGATGGTGATGGGATTTC

Supplementary Table S3. Primers used for *KEAP1* multiplex PCR

Pool	Pair	Target Region		5'-3' sequence
**1**	1	Exon2	F	ACACGACGCTCTTCCGATCTAGAGGTGGTGGTGTTGCTTATCTTC
			R	GACGTGTGCTCTTCCGATCTAAAGGCCTGCTTGGTATGATCC
	3	Exon2	F	ACACGACGCTCTTCCGATCTGCCCAGTTCATGGCCCACAAG
			R	GACGTGTGCTCTTCCGATCTACAACGCTGTCGATCTGGTACA
	5	Exon3	F	ACACGACGCTCTTCCGATCTGGGTGACTGGAGAGTCAGCC
			R	GACGTGTGCTCTTCCGATCTAGCGCCTGGACGTAGAACC
	7	Exon3	F	ACACGACGCTCTTCCGATCTAGCTGCAGAAGTGCGAGATC
			R	GACGTGTGCTCTTCCGATCTTGAGCGACTGTCGGAAGTAG
	9	Exon3	F	ACACGACGCTCTTCCGATCTGGTGGGCGGGCTGTTGTA
			R	GACGTGTGCTCTTCCGATCTCATAGATGTGGCCATCGATGAC
	11	Exon4	F	ACACGACGCTCTTCCGATCTAAGAGTATCTGGCCCTTAAGTATTC
			R	GACGTGTGCTCTTCCGATCTGTGTTCATTGCTGTGATCATTCGC
	13	Exon5	F	ACACGACGCTCTTCCGATCTCAGAGTCACCTTCTCTGCATGGT
			R	GACGTGTGCTCTTCCGATCTATGGGCTAGTCAGGACTCTTCC
**2**	2	Exon2	F	ACACGACGCTCTTCCGATCTCTCCACTGAGTGCAAGGCG
			R	GACGTGTGCTCTTCCGATCTCTTGAAGACAGGGCTGGATGAG
	4	Exon2	F	ACACGACGCTCTTCCGATCTGAGAAGTGTGTCCTCCACGTC
			R	GACGTGTGCTCTTCCGATCTGGTCCTTCTCCTGACACTGCC
	6	Exon3	F	ACACGACGCTCTTCCGATCTCATCAACTGGGTCAAGTACG
			R	GACGTGTGCTCTTCCGATCTAGGGTGAGCTCCTCGAAGA
	8	Exon3	F	ACACGACGCTCTTCCGATCTACAAGCCCACGCAGGTGATGC
			R	GACGTGTGCTCTTCCGATCTCGTCGGGCGAGTTGTTCCTG
	10	Exon3	F	ACACGACGCTCTTCCGATCTCCTGGACTGTTACAACCCCATG
			R	GACGTGTGCTCTTCCGATCTTCAGTGTCTTGGGACTTGCCAGG
	12	Exon4	F	ACACGACGCTCTTCCGATCTGCCTTAATTCAGCTGAGTGTTACTA
			R	GACGTGTGCTCTTCCGATCTAGAGAGAGAAGCTTGGACTCTATCA
	14	Exon6	F	ACACGACGCTCTTCCGATCTGTGACAGGTGGTGACCATCC
			R	GACGTGTGCTCTTCCGATCTAATGATACTCCCCATTGGACTG

Supplementary Table S4. Primers used for *NRF2* multiplex PCR

**Pool**	**Pair**	**Target Region**		**5'-3' sequence**
**1**	1	Exon1	F	ACACGACGCTCTTCCGATCTCGTGTAGCCGATTACCGAGTG
			R	GACGTGTGCTCTTCCGATCTGCCGCGGTTCCCTAGCT
	3	Exon2	F	ACACGACGCTCTTCCGATCTCAGTCAGCGACGGAAAGAGTA
			R	GACGTGTGCTCTTCCGATCTCAGTGTTTCCTTAAACCTGCCATAA
	5	Exon4	F	ACACGACGCTCTTCCGATCTTTTGTTTTGTAGTGGTGCCTTAGAG
			R	GACGTGTGCTCTTCCGATCTCCTGTAACTCAGGAATGGATAATAG
	7	Exon5	F	ACACGACGCTCTTCCGATCTAAGCCTGAAGATAATGTGGGTAGG
			R	GACGTGTGCTCTTCCGATCTTGCTGCTGAAGGAATCCTCAAAAG
	9	Exon5	F	ACACGACGCTCTTCCGATCTACAGTCAACACAGATTTTGGTGATG
			R	GACGTGTGCTCTTCCGATCTCATAGCTGGAAGATTCCACTGAGT
	11	Exon5	F	ACACGACGCTCTTCCGATCTGTACAACCCTTGTCACCATCTCAG
			R	GACGTGTGCTCTTCCGATCTGTTGAGCTTCATTGAACTGCTCTTT
	13	Exon5	F	ACACGACGCTCTTCCGATCTAAGGAGAAAATGACAAAAGCCTTCA
			R	GACGTGTGCTCTTCCGATCTGAGCATTTCACATCACAGTAGGAGC
**2**	2	Exon2	F	ACACGACGCTCTTCCGATCTCCATCAACAGTGGCATAATGTGAAT
			R	GACGTGTGCTCTTCCGATCTGTTGTAACTGAGCGAAAAAGGCTT
	4	Exon3	F	ACACGACGCTCTTCCGATCTCTTGGCAATGGAATATTTAACCATT
			R	GACGTGTGCTCTTCCGATCTTAAATGGAGATTCATTGACGGGACT
	6	Exon4	F	ACACGACGCTCTTCCGATCTCCTGATATTCCCGGTCACATCG
			R	GACGTGTGCTCTTCCGATCTGTTAATAGCACCCTCCAATCCTTCC
	8	Exon5	F	ACACGACGCTCTTCCGATCTGAAGCCAAACTGACAGAAGTTGACA
			R	GACGTGTGCTCTTCCGATCTAATGGCTTAAAGTAGCAGGTGAGGG
	10	Exon5	F	ACACGACGCTCTTCCGATCTCAATGATTCTGACTCCGGCATTT
			R	GACGTGTGCTCTTCCGATCTCAATTCTTTCTCTGGTGTGTTCTCA
	12	Exon5	F	ACACGACGCTCTTCCGATCTATTAACCTCCCTGTTGTTGACTTCA
			R	GACGTGTGCTCTTCCGATCTACTTCGAGATATAAGGTGCTGAGTT
	14	Promoter	F	ACACGACGCTCTTCCGATCTGACCACTCTCCGACCTAAAGG
			R	GACGTGTGCTCTTCCGATCTACCGTGCCACTGCCAAC

Supplementary Table S5. Primers used for Sanger sequencing

Human gene		5'-3' sequence
KEAP1	F	GCCTCACCAAGGACGTAGAT
	R	AGGTCTCCCTCAAGGAGGTG
NRF2	F	ACTGAACTCATCAGGAGGCTG
	R	AAACATGAGCTCTCTCCTTCC

Supplementary Table S6. Upregulated pathways of Hallmark gene set in 2CR vs 2P

Upregulated pathways (2CR vs 2P)	NES	p-val
OXIDATIVE_PHOSPHORYLATION	2.08	0.00
ADIPOGENESIS	2.05	0.00
FATTY_ACID_METABOLISM	1.84	0.00
ESTROGEN_RESPONSE_EARLY	1.64	0.00
BILE_ACID_METABOLISM	1.60	0.00
PEROXISOME	1.58	0.00
MYOGENESIS	1.56	0.00
REACTIVE_OXYGEN_SPECIES_PATHWAY	1.53	0.02
CHOLESTEROL_HOMEOSTASIS	1.50	0.01
ESTROGEN_RESPONSE_LATE	1.49	0.00
UV_RESPONSE_UP	1.48	0.00
EPITHELIAL_MESENCHYMAL_TRANSITION	1.44	0.00
HEME_METABOLISM	1.40	0.01
HYPOXIA	1.32	0.03
XENOBIOTIC_METABOLISM	1.30	0.04
KRAS_SIGNALING_DN	1.24	0.10
COAGULATION	1.20	0.14
MYC_TARGETS_V1	1.17	0.14
NOTCH_SIGNALING	1.16	0.24
UV_RESPONSE_DN	1.11	0.22
IL2_STAT5_SIGNALING	1.10	0.25
HEDGEHOG_SIGNALING	1.08	0.33
COMPLEMENT	1.05	0.35
SPERMATOGENESIS	1.05	0.35
ANGIOGENESIS	1.04	0.40
MTORC1_SIGNALING	1.01	0.42
MYC_TARGETS_V2	1.01	0.44
INTERFERON_ALPHA_RESPONSE	0.97	0.50
APOPTOSIS	0.97	0.53
ANDROGEN_RESPONSE	0.97	0.52
INTERFERON_GAMMA_RESPONSE	0.95	0.59
DNA_REPAIR	0.90	0.72
G2M_CHECKPOINT	0.79	0.96

Supplementary Table S7. Upregulated pathways of Hallmark gene set in 3CR vs 3P

Upregulated pathways (3CR vs 3P)	NES	p-val
INTERFERON_ALPHA_RESPONSE	1.83	0.00
TNFA_SIGNALING_VIA_NFKB	1.82	0.00
INTERFERON_GAMMA_RESPONSE	1.80	0.00
XENOBIOTIC_METABOLISM	1.54	0.00
KRAS_SIGNALING_UP	1.52	0.00
INFLAMMATORY_RESPONSE	1.42	0.01
ALLOGRAFT_REJECTION	1.42	0.02
IL6_JAK_STAT3_SIGNALING	1.29	0.08
REACTIVE_OXYGEN_SPECIES_PATHWAY	1.24	0.16
COMPLEMENT	1.17	0.14
UV_RESPONSE_DN	1.12	0.18
APOPTOSIS	1.11	0.21
IL2_STAT5_SIGNALING	1.08	0.24
HEME_METABOLISM	0.98	0.51
FATTY_ACID_METABOLISM	0.96	0.56
GLYCOLYSIS	0.95	0.61
BILE_ACID_METABOLISM	0.95	0.56
TGF_BETA_SIGNALING	0.84	0.78
UNFOLDED_PROTEIN_RESPONSE	0.77	0.93

Supplementary Table S8. Upregulated pathways of Hallmark gene set in 6CR vs 6P

Upregulated pathways (6CR vs 6P)	NES	p-val
REACTIVE_OXYGEN_SPECIES_PATHWAY	2.33	0.00
TNFA_SIGNALING_VIA_NFKB	2.06	0.00
XENOBIOTIC_METABOLISM	2.04	0.00
OXIDATIVE_PHOSPHORYLATION	1.93	0.00
FATTY_ACID_METABOLISM	1.91	0.00
KRAS_SIGNALING_UP	1.90	0.00
UNFOLDED_PROTEIN_RESPONSE	1.86	0.00
MYC_TARGETS_V1	1.82	0.00
ADIPOGENESIS	1.81	0.00
MTORC1_SIGNALING	1.76	0.00
CHOLESTEROL_HOMEOSTASIS	1.71	0.00
IL2_STAT5_SIGNALING	1.65	0.00
GLYCOLYSIS	1.62	0.00
HEDGEHOG_SIGNALING	1.58	0.01
ESTROGEN_RESPONSE_LATE	1.43	0.01
IL6_JAK_STAT3_SIGNALING	1.40	0.05
UV_RESPONSE_UP	1.38	0.03
APOPTOSIS	1.37	0.01
ANDROGEN_RESPONSE	1.32	0.06
COMPLEMENT	1.30	0.05
TGF_BETA_SIGNALING	1.25	0.10
BILE_ACID_METABOLISM	1.21	0.14
APICAL_SURFACE	1.20	0.19
P53_PATHWAY	1.18	0.07
HEME_METABOLISM	1.17	0.10
MYC_TARGETS_V2	1.17	0.18
PEROXISOME	1.15	0.16
PROTEIN_SECRETION	1.08	0.30
PI3K_AKT_MTOR_SIGNALING	1.05	0.33
ANGIOGENESIS	1.02	0.42
G2M_CHECKPOINT	1.00	0.42
NOTCH_SIGNALING	0.94	0.55
E2F_TARGETS	0.54	1.00

Supplementary Table S9. Clinical features of 31 HNSCC patients evaluated by IHC

No.	Age	Sex	Primary site	Clinical Stage	NRF2 IHC score
1	60	M	Hypopharynx	4a	84.72
2	76	F	Tongue	4a	103.32
3	70	M	Tongue	4a	82.61
4	68	M	Tongue	4a	82.08
5	74	M	Tongue	4a	105.11
6	60	M	Tongue	4a	101.84
7	75	M	Maxillary Gingiva	4a	82.38
8	71	M	Salivary Grand	4a	98.89
9	53	M	Hypopharynx	4a	79.58
10	65	M	Lip	4a	73.53
11	66	M	Hypopharynx	4a	116.89
12	61	M	Hypopharynx	4a	101.22
13	56	F	Maxillary Gingiva	4a	59.81
14	59	F	Floor of Mouth	4a	63.29
15	70	M	Floor of Mouth	4a	154.79
16	64	M	Oropharynx	4a	72.65
17	75	F	Hypopharynx	4b	109.12
18	54	M	Salivary Grand	4a	77.62
19	54	F	Tongue	4a	113.15
20	76	M	Mandibular Gingiva	4a	77.72
21	61	M	Tongue	4a	140.15
22	60	M	Maxillary Gingiva	4a	44.57
23	78	M	Larynx	4a	0.00
24	45	M	Tongue	4a	55.25
25	68	M	Hypopharynx	4a	120.48
26	27	F	Tongue	4a	185.71
27	78	M	Hypopharynx	4a	185.95
28	78	M	Hypopharynx	4a	47.29
29	83	M	Hypopharynx	4a	98.08
30	54	M	Tongue	4a	134.33
31	52	M	Maxillary Gingiva	4a	15.38

Supplementary Figure S1

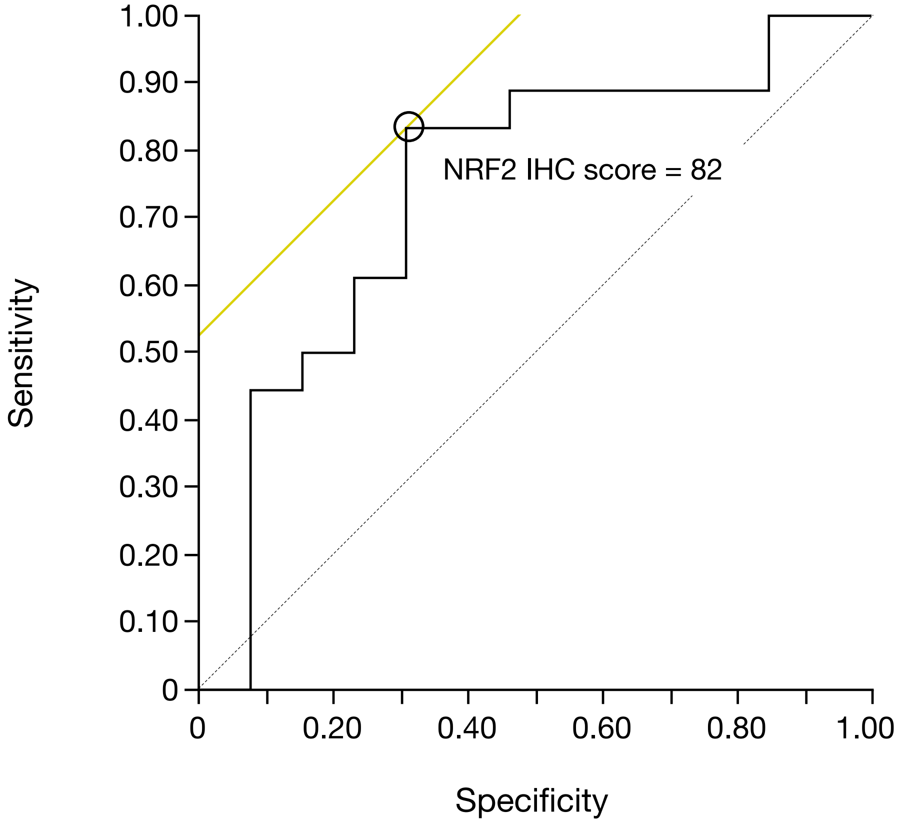

Supplementary Figure S1. Receiver operating characteristic (ROC) curve analysis for determining the optimal cutoff of NRF2 IHC score.

The cutoff value for NRF2 IHC score was calculated based on the ROC curve. The black curve represents the ROC curve, and the dotted diagonal line represents the reference line with a slope of 1 passing through the origin. The y-axis represents sensitivity, and the x-axis represents 1 – specificity. The yellow line indicates the tangent line to the ROC curve with a slope of 1, and the point of tangency corresponds to the optimal cutoff value for the NRF2 IHC score.

Supplementary Figure S2

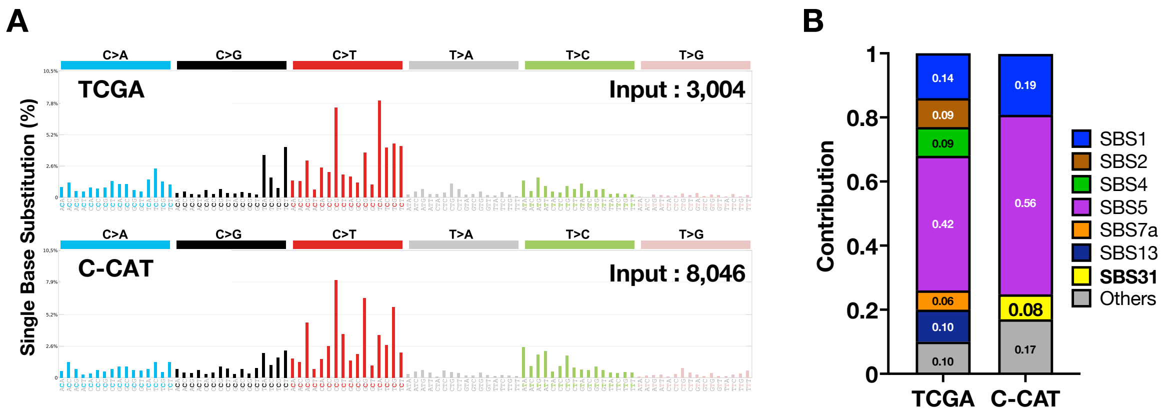

Supplementary Figure S2. Mutational signature analysis in HNSCC patients from the TCGA and C-CAT datasets.

A Mutational spectrum of single base substitutions (SBSs) in HNSCC from the TCGA (top panel) and C-CAT (bottom). **B** Contribution of SBS signatures identified in **(**A). Note that SBS31, associated CDDP treatment, was observed only in the C-CAT dataset.

Supplementary figure S3

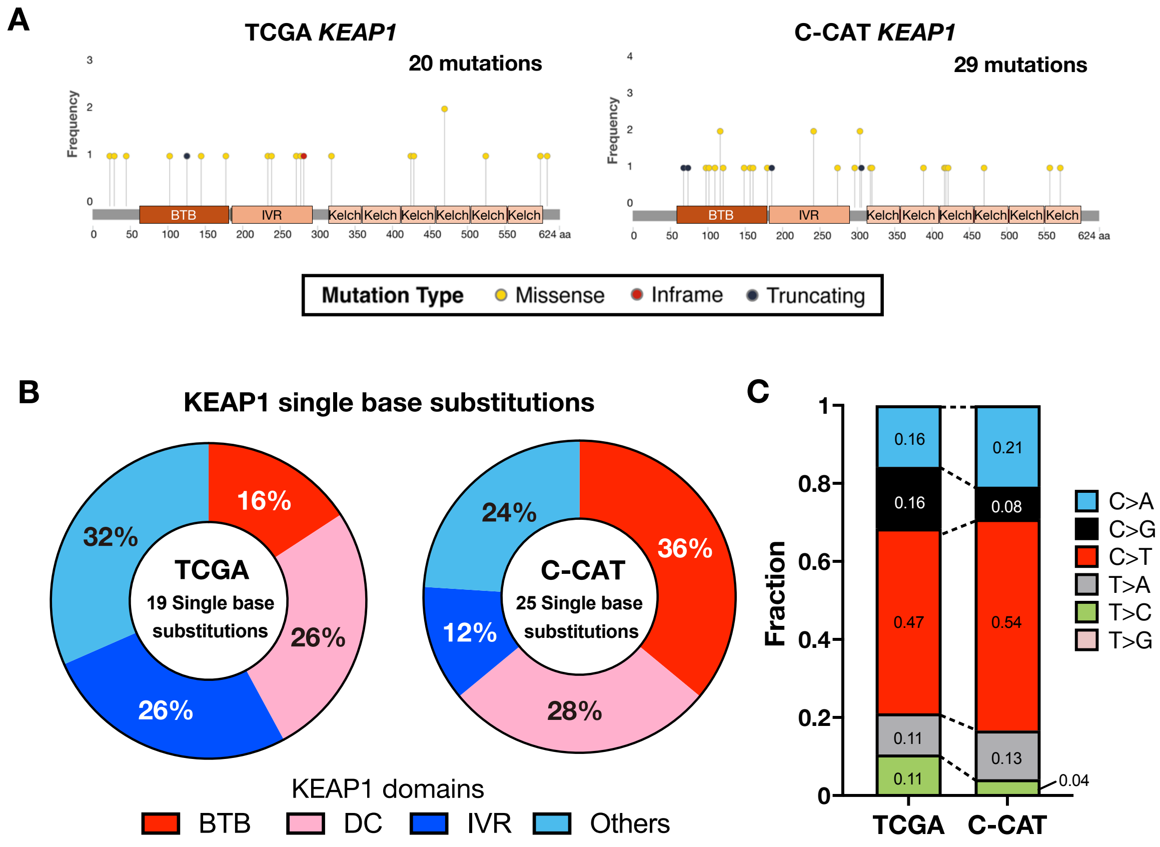

Supplementary Figure S3. Mutational analysis in of *KEAP1* in HNSCC patients from the TCGA and C-CAT datasets.

A Lollipop plots showing genetic variants *KEAP1* in the TCGA (left panel) and C-CAT datasets (right panel). **B** Distribution of mutations within functional domains of KEAP1 in TCGA and C-CAT datasets. **C** Proportion of single base substitutions in the *KEAP1* gene in the TCGA and C-CAT datasets.

Supplementary figure S4

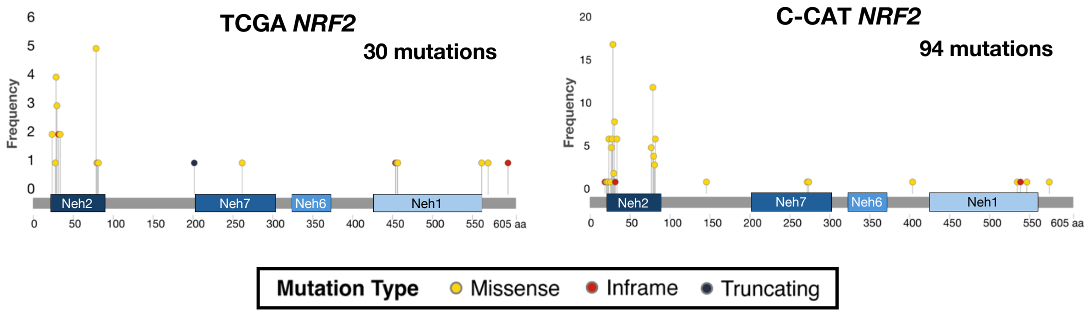

Supplementary Figure S4. Mutational analysis in of *NRF2* in HNSCC patients from the TCGA and C-CAT datasets.

Lollipop plots showing genetic variants *NRF2* in the TCGA (left panel) and C-CAT datasets (right panel).

References

[1] Maruyama, A., Tsukamoto, S., Nishikawa, K., Yoshida, A., Harada, N., Motojima, K. *et al.* Nrf2 regulates the alternative first exons of CD36 in macrophages through specific antioxidant response elements. *Arch Biochem Biophys* **477**, 139-145 (2008).

[2] Watai, Y., Kobayashi, A., Nagase, H., Mizukami, M., McEvoy, J., Singer, J. D. *et al.* Subcellular localization and cytoplasmic complex status of endogenous Keap1. *Genes Cells* **12**, 1163-1178 (2007).
